# Serum Cytokine and miRNA Levels Are Differently Expressed in Right- and Left-Sided Colon Cancer

**DOI:** 10.3390/jcm12185986

**Published:** 2023-09-15

**Authors:** Valentina De Nunzio, Rossella Donghia, Pasqua L. Pesole, Sergio Coletta, Nicola Calò, Maria Notarnicola

**Affiliations:** National Institute of Gastroenterology-IRCCS “Saverio de Bellis”, Castellana Grotte, 70013 Bari, Italy; valentina.denunzio@irccsdebellis.it (V.D.N.); rossella.donghia@irccsdebellis.it (R.D.); letizia.pesole@irccsdebellis.it (P.L.P.); sergio.coletta@irccsdebellis.it (S.C.); nicola.calo@irccsdebellis.it (N.C.)

**Keywords:** circulating cytokines, miRNA, colorectal cancer, tumor site

## Abstract

The tumor location in colorectal cancer (right- or left-sided colon cancer) is a key factor in determining disease progression. Right- and left-sided colon tumors are different in their clinical and molecular characteristics. Dysregulation of serum levels of proinflammatory cytokines, such as Transforming Growth Factor β (TGF-β) and Tumor Necrosis Factor-α (TNF-α), and Peroxisome Proliferator Activated Receptor-γ (PPAR-γ), known to be a growth-limiting and differentiation-promoting factor, as well as changes in miRNAs expression, are the major signaling pathways involved in the pathogenesis of this neoplasia. In the serum from 60 colorectal cancer (CRC) patients, we compared the differences in the expression of the levels of TGF-β, TNF-α, and PPAR-γ and in the expression of the main human miRNAs between right and left CRC. A significant over-expression in the TGF-β and TNF-α levels was observed in the serum from right-sided colon cancer patients. For the PPAR-γ, the patients with CRC located on the right-side showed lower levels than those detected in the serum from left-sided CRC subjects. Furthermore, significant differences also existed in the expression of specific circulating miRNAs between right- and left-sided CRC. In particular, the right upregulated miRNAs were all involved in the cell growth and proliferation related pathways. These findings confirm that the analysis of circulating levels of TGF-β, TNF-α, and PPAR-γ, as well as the study of the specific miRNAs in the serum, are able to identify specific characteristics of CRC patients, useful for choosing a personalized treatment protocol.

## 1. Introduction

Colorectal cancer (CRC) is the second leading cause of cancer-associated mortality worldwide and the most common cancer of the gastro-intestinal tract [1]. CRC can be divided in right and left-sided CRC. Right- sided tumors form in cecum, ascending colon and transverse colon, while the left-sided tumors are located at the descending colon, sigma and rectum [2,3]. Epidemiological studies show that the incidence of right- or left- sided CRC can be influenced by sex and age with implications related to survival [4]. Right-sided (proximal) colon cancer is a more aggressive-type tumor compared to left-sided (distal) colon cancer [5], and patients with proximal colon cancer are more often females than males [6]. In an advanced stage of the neoplasia, proximal colonic tumors are more often flat, while distal colonic tumors are a polypoid-type [7].

CRC exhibits also different molecular and pathological characteristics depending on tumor location. Chromosomal instability and p53 mutations are more often observed in left-sided colon cancer [8], whereas microsatellite instability (MSI) and B-Raf proto-oncogene, serine/threonine kinase (BRAF) mutation are often observed in right-sided colon cancer [9,10]. 

Therefore, early diagnosis of the neoplastic lesion and identification of the best protocol to treat patients with CRC are the main objectives to reduce the incidence of mortality from this type of tumor [11,12].

Several clinical studies have suggested that inflammatory tissue conditions promote cancer development; in particular, some evidence pointed out a relationship between the activation of inflammatory cytokines and a poor prognosis in patients with CRC [13].

Impaired regulation of serum levels of proinflammatory cytokines, such as Transforming Growth Factor β (TGF-β) and Tumor Necrosis Factor Alpha (TNF-α), and the down-expression of Peroxisome Proliferator Activated Receptor Gamma (PPAR-γ), known to be a growth-limiting and differentiation-promoting factor, are major signaling pathways involved in the pathogenesis of CRC [13,14].

TGF-β is an inflammatory cytokine that regulates tissue growth and elevated levels of TGF-β are connected to advanced CRC stages [15]. TGF-β acts as a tumor suppressor by inhibiting proliferation and inducing apoptosis during the early stages of tumorigenesis [16], whereas it is considered a tumor promoter in advanced CRC.

TGF-β promotes the immune escape of CRC cells through the accumulating mutations in the TGF-β signaling cascades [17], including the mutation in genes that encode TGF-β ligands [18,19]. 

The serum cytokine TNF-α is considered a biomarker associated with a clinical outcome in CRC patients [20]. TNF-α is involved in maintenance and homeostasis of the immune system, inflammation and host defense. This versatile cytokine has a central role in apoptosis, angiogenesis, and immune cells’ recruitment and regulation [20,21]. Recently, the inhibition of endogenous TNF-α is a standard of care for chronic inflammatory diseases, such as ulcerative colitis, Crohn’s disease, and several other diseases, including CRC [22].

Genetic and epigenetic modifications of the PPAR-γ signaling-related pathways play a fundamental role in the initiation and progression of CRC [23]. PPAR-γ exerts a tumor suppressor role in cancer, potentially linked with the Wnt/β catenin pathway. Dysregulation in PPAR-γ expression has been shown to promote tumorigenesis in the colon, and the efforts to design PPAR-γ agonists could be beneficial in therapy and/or prevention for patients with CRC [23,24,25].

Epidemiological studies have widely demonstrated that tumor location, in the colon, is a crucial factor influencing disease management [26]. Right- and left-sided colon tumors are different in their clinical and molecular characteristics [27], due to their distinct embryological origins. Also, the microenvironment can influence the development of the tumor site due to the dynamic exchanges between the cancer cells and their neighboring cells [28], as well as diet, physical activity, and smoking. Right-CRC has been shown to be characterized by a more advanced tumor stage, larger tumor size, and poorly differentiated tumor cells, and affects older and female subjects more than left-sided CRC [29,30]. 

Several studies have demonstrated that differences in clinical outcomes and drug responsiveness are dependent on the location of cancer along the colon [31]. Right-sided colon cancer occurring within the cecum, ascending colon, hepatic flexure or in transverse, are often influenced by diet [31]. A significant increase in the incidence of CRC in eastern populations seem to be due to a change in dietary preferences in favor of a saturated fatty acid-rich western style diet [32]. A high dietary intake of saturated fatty acids has been implicated in obesity-associated gene expression profile and metabolic syndrome [33,34]. Cancer cells rely on the nutrient to maintain their increased demand for energy and, therefore, available nutrients, as well as intestinal microbioma, which regulates cancer cell proliferation and differentiation. 

The human gut microbioma’s function is to protect against infection, and produce vitamins and immune cells [35]. However, under the condition of imbalance or dysbiosis, the gene coding of molecules is broken, and harmful compounds are metabolized by pathogenic microorganisms. Recently, significant differences were observed in colon microbioma between right- and left-sided colon cancer [36]. The heterogeneous nature of the colon microbiome correlated with some clinicopathologic features of CRC, including tumor site [37]. 

The gut microbiome appears to interact with host microRNAs (miRNAs), which in turn are able to shape the composition and metabolism of gut microbes [38]. In addition, it is known that some species of bacteria facilitate tumorigenesis in CRC, in a miRNA-dependent manner [39].

miRNAs are short, non-coding RNA sequences of 19 to 23 nucleotides able to regulate the expression of genes involved in both normal healthy conditions and in the pathogenesis of several diseases, including cancer [40].

miRNA profiling has been shown to be a potential biomarker in site-specific CRC [41], as different miRNAs regulate cell growth, proliferation and metastasis in right versus left CRC [42]. Eneh et al. demonstrated that miR-30, miR-155, miR-150, miR-31, miR-330, miR-15 and miR-130 are upregulated in right-sided CRC compared to left-sided CRC [42]. Also, Mjell et al. showed that there are miRNAs, such as miR-615-3p, which are upregulated in right-sided colon cancer compared to left-sided cancers; however, they also found that this miRNA is upregulated in the right-sided normal colon compared to the left-sided normal colon. The difference in expression of these miRNAs between the right and left side is probably due to biological differences between the left and right colon [43], whereas let-7, miR-193, miR-145, miR-196, miR-9 and miR-99 are upregulated in left-sided CRC compared to right-sided CRC [42].

The tumor location may be indicative for the activation of predominant molecular pathways participating in the progression of the neoplasia. miRNAs detected in the right-sided CRC have been demonstrated to participate in the TGF-β signaling pathways [44], whereas the left miRNAs participate in the mTor, Wnt, and P13K-Akt signaling pathways [44,45]. 

To date, CRC has no specific serum biomarkers to promote an early diagnosis and a targeted treatment. Recently, it is demonstrated that differences in TGF-β pathways were able to stratify CRC into two clusters exhibiting significant differences in survival outcomes [4]. In this context, changes in the serum levels of a panel of circulating cytokines was detected in CRC patients [46], but the mechanism involving these molecules remains to be determined. 

Assuming the involvement of circulating cytokines, such as TGF-β, TNF-α, and PPAR-γ, in metabolic pathways related to CRC, in this study, we compared the differences in the expression of serum levels of these cytokines between right- and left-sided CRC.

Moreover, since tissue-based studies have found several differentially expressed miRNAs in the cascade of colon carcinogenesis, here, we have also investigated the expression of the main human miRNAs in relation to tumor site.

## 2. Materials and Methods

### 2.1. Patients

The study was conducted on serum samples collected in the Biobank of our Institute and taken from 60 patients (42 males and 18 females, mean age 68.8 ± 8.8) with histologically proven primary CRC. None of the patients received preoperative chemotherapy and/or radiotherapy. Written informed consent was obtained from all the patients for blood sample storage and clinical data collection.

Primary tumors located at the cecum, ascending colon, and transverse colon were defined as right-sided colon cancer, whereas primary tumors located at the splenic flexure, descending colon, sigmoid and rectum colon were defined as left-sided colon cancer.

### 2.2. Biochemical Analysis

Serum levels of TGF-β, TNF-α and PPAR-γ were performed with a quantitative sandwich ELISA kit (MyBioSource Inc., San Diego, CA, USA) according to the manufacturer’s recommendations. Briefly, for each cytokine, standards and serum samples were pipetted in duplicate into a 96-well plate together with the specific antibody. A HRP conjugate reagent was added and the plate was incubated at 37 °C. Next, the wells were washed, the substrate reagent was added, and after 15 min at 37 °C, the color changed to blue. The enzyme–substrate reactions were terminated by the addition of the stop solution. The optical density (OD) was read at 450 nm with a plate reader, and the concentration of TGF-β, TNF-α and PPAR-γ was calculated by comparing the OD of the sample with each standard curve for single cytokine.

### 2.3. miRNAs Analysis

The analysis of serum human miRNAs was performed by Real-Time quantitative PCR. Total RNA was isolated from 200 μL of serum using the miRNeasy Serum/Plasma Advanced Kit (Qiagen, Hilden, Germany), following the manufacturer’s instructions and previously described [47]. Briefly, buffer RPL and buffer RPP were added to serum, incubated at room temperature and centrifuged at 10,000 rpm × 3 min. The supernatant was recovered, placed on a RNeasy UCP MinElute column with the addition of 1 volume of isopropanol, centrifuged and then flow-through was discarded. The following buffers were then added (buffer RWT, buffer RPE and 80% ethanol). After addition, each solution was centrifuged at 10,000 rpm and flow-through was discarded. The RNeasy UCP MinElute column was again centrifuged at full speed for 5 min to dry the membrane and 20 μL of RNase-free water was added directly to the center of the spin column membrane, incubated for 1 min and centrifuged at full speed for 1 min to elute RNA. To monitor RNA isolation/purification 1 μL of RNA Spike-in mix (Quiagen, Hilden, Germany) containing UniSp2, UniSp4, UniSp5 was added, while 1 μL of UniSp6 and cel-miR-39-3p to evaluate the efficiency of the reverse transcription step. 

The RNA obtained was reverse transcribed using the miRCURY LNA RT Kit (Quiagen, Hilden, Germany) following the manufacturer’s instructions. After the reverse transcription step, the quantification of the main circulating human miRNAs was performed by miRCURY LNA SYBR Green PCR Kit (Qiagen, Hilden, Germany), containing a high-performance PCR master mix, and the miRCURY LNA miRNA Serum/Plasma Focus PCR Panel (Qiagen, Hilden, Germany, Cat. no. YAHS-106Y). The Qiagen PCR Panel contained 179 circulating miRNAs, including potential reference genes (miR103-3p, miR-191-5p, miR-423-5p, miR-93-5p, and miR-425-5p) and controls for hemolysis (miR-451a and miR-23a-3p). The data analysis web portal (http://www.qiagen.com/geneglobe, accessed on 28 June 2023) calculates fold change using the Δ-ΔCT method, in which ΔCT is calculated between the miRNA of interest and an average of the reference miRNAs, followed by Δ-ΔCT calculations. The analysis report was exported from the Qiagen web portal GeneGlobe.

### 2.4. Statistical Analysis

Patients’ characteristics are reported as mean and standard deviation (Mean ± SD) for continuous variables, and as frequency and percentages (%) for categorical. To test the association between the independent groups (Right side vs. Left side), Chi-square or Fisher test was used for categorical variables, where necessary, and the Wilcoxon Rank Mann–Whitney was chosen for continuous variables. The heatmap was generated based on complete linkage of hierarchical clustering with Euclidean distance.

To test the null hypothesis of non-association, the two-tailed probability level was set at 0.05. The analyses were conducted using StataCorp.2021 software (Release 17) College Station, TX, USA: StataCorp LLC while RStudio (“Mountain Hydrangea” Release) was used for the plots.

CT values of miRNAs analysis were exported to an Excel table, the table was uploaded to the Data Analytics web portal at http://www.qiagen.com/geneglobe, accessed on 28 June 2023.

### 2.5. Bioinformatics Analysis

Functional analysis of these selected miRNAs was carried out with the novel release 2.0 of the miRNA Pathway Dictionary Database (miRPathDB) that is freely accessible at https://mpd.bioinf.uni-sb.de/, accessed on 29 June 2023 [48]. 

miRPathDB is a database that provides information on miRNAs, their target genes, and their target pathways. In this study, the results for the analyzed miRNA targets were represented as a heat map by selecting the KEGG (Kyoto Encyclopedia of Genes and Genomes) database, and “experimental (strong)” evidence, at least one of the significant pathways for miRNA, and at least one significant miRNA for the pathway. In the heat map, the color of individual entries corresponds to the *p*-value of the associated enrichment result (darker colors indicate more significant enrichments of miRNA target genes in the corresponding biological processes).

## 3. Results

The comparison of histopathological and blood parameters in patients with right- and left-sided colon cancer is shown in Table 1. 

**Figure 1:**
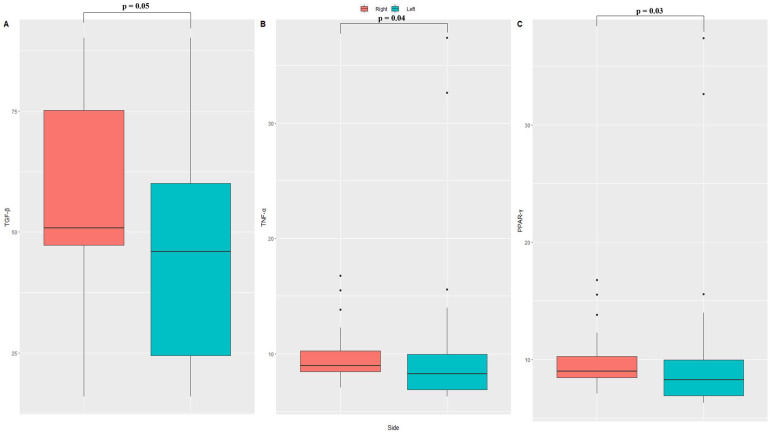
Distribution of cytokines in right- and left-sided CRC. (**A**) Serum levels of TGF-β (Transforming Growth Factor Beta); (**B**) Serum levels of TNF-α (Tumor Necrosis Factor Alpha); (**C**) Serum levels of PPAR-γ (Peroxisome Proliferator Activated-γ). Wilcoxon rank-sum test was used to compare cytokines levels median in different sides.

No differences were observed for the variables, such as age, sex, and tumor characteristics between the two categories of the patients. However, the different biology of right- and left-sided colon tumors was confirmed by a different expression of circulating molecules involved in the regulation and control of cell proliferation. In this regard, a statistically significant increase in circulating levels of TGF-β and TNF-α from patients with CRC located to right side was observed when compared with the patients with the colon tumor located at left side (Figure 1). For the PPAR-γ, the right-side CRC patients showed lower levels than those detected in the serum from left-sided colon cancer patients (Figure 1). These findings confirm that left- and right-sided colon cancers harbor distinct and diverse characteristics. 

The identification of the circulating cytokines profile, capable to recognize specific subgroups of CRC patients, allow an appropriate clinical treatment, improving the efficacy of therapies.

**Figure 2:**
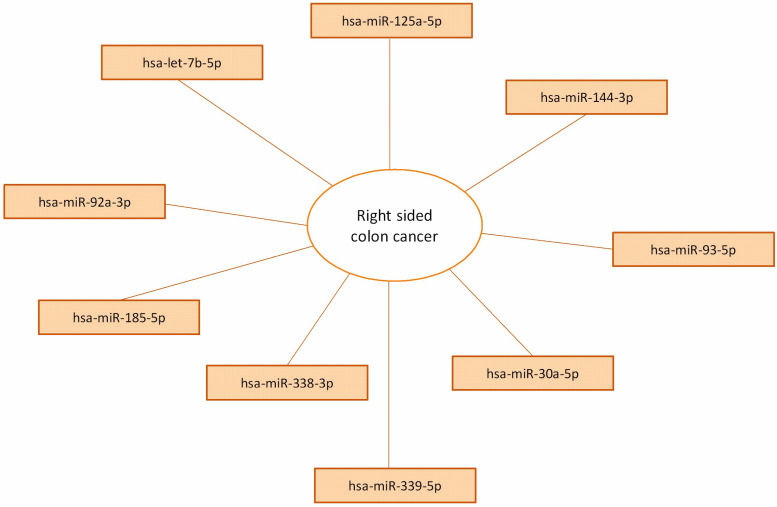
Upregulated miRNAs detected in the serum of right-sided CRC patients.

The analysis of circulating miRNAs allowed to identify further differences between right- and left-sided colon tumor patients. In particular, after excluding several miRNA targets with Ct values > 35 for their low expression, a panel of nine serum miRNAs (hsa-miR-125a-5p, hsa-miR-144-3p, hsa-miR-93-5p, hsa-miR-30a-5p, hsa-miR-339-5p, hsa-miR-338-3p, hsa-miR-92a-3p, hsa-let-7b-5p, hsa-miR-185-5p) distinguished right- and left-sided CRC with fold changes > 2 (Figure 2). All these nine miRNAs resulted in upregulating the serum from patients with right-sided CRC, compared with left-sided CRC patients. 

**Figure 3:**
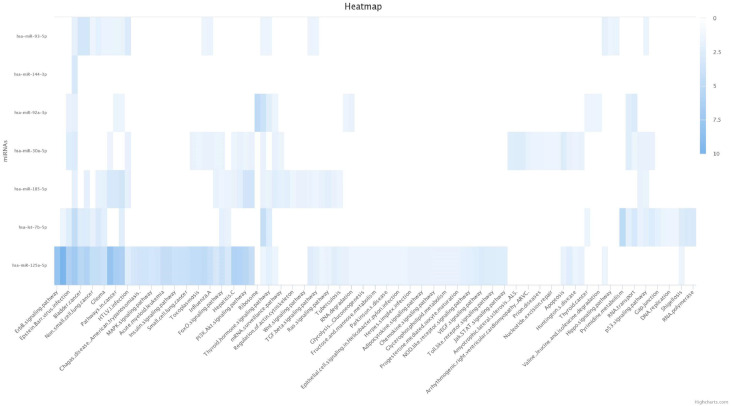
Heatmap plot of significant KEGG pathways for 7 miRNAs. Rows represent the enrichment results for the targets of the 7 miRNAs. Columns represent all KEGG pathways that are significant for the different miRNAs. The color of individual fields represents the −log10-transformed *p*-value of the respective enrichment results. Darker colors indicate more significant associations between miRNA and target pathway.

The heatmap, shown in Figure 3, depicts the enrichment results of seven out of the nine miRNAs in the Kyoto Encyclopedia of Genes and Genomes (KEGG) database. Two of these seven miRNAs, miR-125a-5p and let-7b-5p, were involved in the regulation of a great number of pathways and with more significant associations. The hsa-miR-125a-5p seems to be mainly involved in cancer-related pathways, whereas the hsa-let-7b-5p seems to participate in the DNA replication and cell metabolism pathways.

## 4. Discussion

Biologically, CRC is a heterogeneous type of tumor, characterized by a significative clinical variability [49]. In colon cancer, the site of the tumor is one aspect of heterogeneity, due to distinct biological and molecular features existing between the left and right colon tract. 

Several clinical studies have demonstrated that left- and right-sided colon tumors are different diseases, based also on their surgical procedures, as well as complication rates and recurrence patterns [50]. In particular, right-sided colon cancers are characterized by high immunogenicity and a worse prognosis [3,51]. Therefore, different treatment strategies should be applied for patients based on the location of the neoplasia.

It is widely recognized that serum cytokine content reflects what is present in the tumor microenvironment [46]. Alterations in the expression of cytokines and/or chemokines secreted in the tumor microenvironment are key events involved in the pathogenesis of CRC. Immune mediators’ changes are correlated with tumor progression and prognosis of this type of cancer [52]. 

On the basis of these premises, tumor microenvironment-derived serum markers are considered a rapid and easily accessible diagnostic and prognostic tool for CRC patients’ outcome [53]. 

Interestingly, TGF-β and TNF-α stimulate cell growth and invasion in an autocrine and paracrine manner. On tumor progression, TGF-β facilitates tumor angiogenesis and metastasis [54], as well as TNF-α promoting the necrosis of endothelial cells involved in tumor cells migration [55]. 

PPAR-γ is one of the three different isoforms of the PPARs family (PPARα, PPARβ/δ and PPAR-γ) and acts as a tumor suppressor in CRC. Evidence that PPAR-γ has an antitumor effect in CRC comes from several experimental studies showing that PPAR-γ activation is associated with the inhibition of cell growth. Moreover, PPAR-γ also has a direct inhibitory effect on the expression of proinflammatory cytokines, including TNF-α [23,56]. 

Increased serum levels of TGF-β and TNF-α, detected in the patients with right-sided colon cancer, could predict adverse outcome with respect to the patients with left-sided colon cancer. These differences in survival outcomes are principally due to an altered organization of extracellular matrix observed in right-sided CRC [57,58]. 

Moreover, the down-regulation of the PPAR-γ levels observed in the serum from right-sided colon cancer patients confirms the loss of the PPAR-γ modulators with an inhibitory effect on colon cancer cell proliferation. In this context, PPAR-γ signaling is drawing increasing attention as a key molecule involved in CRC pathogenesis. The tumor suppressive activity of PPAR-γ could be altered at multiple levels through abnormal DNA phosphorylation and hypermethylation, and presumably could be related to patients’ prognoses [23]. 

Recently, the interaction between the tumor and the immune cells has been detected to be modulated by small proteins, as the circulating cytokines TGF-β, TNF-α, and PPAR-γ are detectable in peripheral blood [59].

Significant differences also exist in the expression of several circulating miRNAs between the two anatomical sites of CRC. By comparing the miRNAs expression between right- and left-sided CRC, we detected nine upregulated miRNAs in the serum of patients with right-sided CRC. The predominant biological pathways involving these upregulated miRNAs are mainly related to cell growth and proliferation. Interestingly, hsa-miR-93-5p and especially hsa-miR-125a-5p participate in the TGF-β signaling pathway. This finding is consistent with other studies showing that the TGF-β pathway is predominant in right-sided CRC [42]. The regulation of the TGF-β pathway by specific miRNAs expression might play a key role in tumor aggressiveness, facilitating an inflammatory reaction via a TNF receptor family mechanism [46]. 

Aberrant expression of miRNAs has been shown to play a role in CRC initiation and progression. Several miRNAs can act as oncogenes by inhibiting the expression of tumor suppressor genes and, on the other hand, can also exhibit tumor-inhibiting characteristics that favor the control of cell proliferation [38].

A close link between specific miRNAs and CRC development exists, with changes in miRNAs expression particularly noticeable during the transition from adenoma to a malignant tumor [60].

Although further clinical and experimental studies will be needed before designating specific miRNAs as potential new biomarkers for CRC, the use of these molecules to establish personalized treatment protocols based on tumor location should be encouraged in CRC management.

## 5. Conclusions

Despite the small sample size, which is considered a limitation of the study, the identification of specific circulating serum cytokines and miRNAs associated with right-sided colon cancer has certain implications for understanding the role and the impact of these molecules in the diagnostic and therapeutic assessment of CRC patients. The potential use of these biomarkers could be a valuable tool for choosing the safest and most effective treatment protocol and to understand how CRC onset influences cancer cell biology and metabolism through specific interactions across cells and the tumor microenvironment. 

## Figures and Tables

**Table 1:** Comparison of epidemiological and blood parameters between patients with left- and right colon cancer (Left *vs.* Right) (*n =* 60).

Parameters *	Total Cohort	Side		*p* ^^^
		Left (*n =* 43)	Right (*n =* 17)	
Gender (M) (%)	42 (70.00)	30 (69.77)	12 (70.59)	0.95 ^ψ^
Age (yrs)	68.80 ± 8.80	68.63 ± 9.39	69.23 ± 7.33	0.87
Tumor Stage (%)				0.67 ^ψ^
I	9 (19.57)	6 (16.67)	3 (30.00)	
II	14 (30.43)	11 (30.56)	3 (30.00)	
III	22 (47.83)	18 (50.00)	4 (40.00)	
IV	1 (2.17)	1 (2.78)	0 (0.00)	
Histological Grade				0.55 ^ψ^
G1	3 (6.25)	3 (9.09)	0 (0.00)	
G2	24 (50.00)	15 (45.45)	9 (60.00)	
G3	21 (43.75)	15 (45.45)	6 (40.00)	
Metastasis (Yes) (%)	6 (15.00)	4 (14.29)	2 (16.67)	0.99 ^ψ^

* As Mean and Standard Deviation for continuous variables, and as frequency and percentage (%) for categorical. ^^^ Wilcoxon rank-sum test (Mann–Whitney), ^ψ^ Chi-Square or Fisher test, where necessary.

## Data Availability

Data are available from corresponding author upon reasonable request.

## References

[1] Siegel R.L., Miller K.D., Fuchs H.E., Jemal A. (2022). Cancer statistics, 2022. CA Cancer J. Clin..

[2] Iacopetta B. (2002). Are there two sides to colorectal cancer?. Int. J. Cancer.

[3] Baran B., Mert Ozupek N., Yerli Tetik N., Acar E., Bekcioglu O., Baskin Y. (2018). Difference Between Left-Sided and Right-Sided Colorectal Cancer: A Focused Review of Literature. Gastroenterol. Res..

[4] Weiss J.M., Pfau P.R., O’Connor E.S., King J., LoConte N., Kennedy G., Smith M.A. (2011). Mortality by stage for right- versus left-sided colon cancer: Analysis of surveillance, epidemiology, and end results—Medicare data. J. Clin. Oncol..

[5] Hansen I.O., Jess P. (2012). Possible better long-term survival in left versus right-sided colon cancer—A systematic review. Dan. Med. J..

[6] Pal S.K., Hurria A. (2010). Impact of age, sex, and comorbidity on cancer therapy and disease progression. J. Clin. Oncol..

[7] Kaku E., Oda Y., Murakami Y., Goto H., Tanaka T., Hasuda K., Yasunaga M., Ito K., Sakurai K., Fujimori T. (2011). Proportion of flat- and depressed-type and laterally spreading tumor among advanced colorectal neoplasia. Clin. Gastroenterol. Hepatol..

[8] Markowitz S.D., Bertagnolli M.M. (2009). Molecular origins of cancer: Molecular basis of colorectal cancer. N. Engl. J. Med..

[9] Jacobs E.T., Thompson P.A., Martinez M.E. (2007). Diet, gender, and colorectal neoplasia. J. Clin. Gastroenterol..

[10] Missiaglia E., Jacobs B., D’Ario G., Di Narzo A.F., Soneson C., Budinska E., Popovici V., Vecchione L., Gerster S., Yan P. (2014). Distal and proximal colon cancers differ in terms of molecular, pathological, and clinical features. Ann. Oncol..

[11] Ferlay J., Colombet M., Soerjomataram I., Dyba T., Randi G., Bettio M., Gavin A., Visser O., Bray F. (2018). Cancer incidence and mortality patterns in Europe: Estimates for 40 countries and 25 major cancers in 2018. Eur. J. Cancer.

[12] Baidoun F., Elshiwy K., Elkeraie Y., Merjaneh Z., Khoudari G., Sarmini M.T., Gad M., Al-Husseini M., Saad A. (2021). Colorectal Cancer Epidemiology: Recent Trends and Impact on Outcomes. Curr. Drug. Targets.

[13] Ding J.T., Zhou H.N., Huang Y.F., Peng J., Huang H.Y., Yi H., Zong Z., Ning Z.K. (2022). TGF-beta Pathways Stratify Colorectal Cancer into Two Subtypes with Distinct Cartilage Oligomeric Matrix Protein (COMP) Expression-Related Characteristics. Biomolecules.

[14] Itatani Y., Kawada K., Sakai Y. (2019). Transforming Growth Factor-beta Signaling Pathway in Colorectal Cancer and Its Tumor Microenvironment. Int. J. Mol. Sci..

[15] Liu S., Ren J., Ten Dijke P. (2021). Targeting TGFbeta signal transduction for cancer therapy. Signal Transduct. Target. Ther..

[16] Hao Y., Baker D., Ten Dijke P. (2019). TGF-beta-Mediated Epithelial-Mesenchymal Transition and Cancer Metastasis. Int. J. Mol. Sci..

[17] Derynck R., Akhurst R.J., Balmain A. (2001). TGF-beta signaling in tumor suppression and cancer progression. Nat. Genet..

[18] Abbona A., Ricci V., Paccagnella M., Granetto C., Ruatta F., Cauchi C., Galizia D., Ghidini M., Denaro N., Merlano M.C. (2023). Baseline Cytokine Profile Identifies a Favorable Outcome in a Subgroup of Colorectal Cancer Patients Treated with Regorafenib. Vaccines.

[19] Ricci V., Granetto C., Falletta A., Paccagnella M., Abbona A., Fea E., Fabozzi T., Lo Nigro C., Merlano M.C. (2020). Circulating cytokines and outcome in metastatic colorectal cancer patients treated with regorafenib. World J. Gastrointest. Oncol..

[20] Laha D., Grant R., Mishra P., Nilubol N. (2021). The Role of Tumor Necrosis Factor in Manipulating the Immunological Response of Tumor Microenvironment. Front. Immunol..

[21] Ji H., Cao R., Yang Y., Zhang Y., Iwamoto H., Lim S., Nakamura M., Andersson P., Wang J., Sun Y. (2014). TNFR1 mediates TNF-alpha-induced tumour lymphangiogenesis and metastasis by modulating VEGF-C-VEGFR3 signalling. Nat. Commun..

[22] Kantola T., Klintrup K., Vayrynen J.P., Vornanen J., Bloigu R., Karhu T., Herzig K.H., Napankangas J., Makela J., Karttunen T.J. (2012). Stage-dependent alterations of the serum cytokine pattern in colorectal carcinoma. Br. J. Cancer.

[23] Sabatino L., Pancione M., Votino C., Colangelo T., Lupo A., Novellino E., Lavecchia A., Colantuoni V. (2014). Emerging role of the beta-catenin-PPARgamma axis in the pathogenesis of colorectal cancer. World J. Gastroenterol..

[24] Michalik L., Desvergne B., Wahli W. (2004). Peroxisome-proliferator-activated receptors and cancers: Complex stories. Nat. Rev. Cancer.

[25] Fredericks E., Dealtry G., Roux S. (2018). Beta-Catenin Regulation in Sporadic Colorectal Carcinogenesis: Not as Simple as APC. Can. J. Gastroenterol. Hepatol..

[26] Lee M.S., Menter D.G., Kopetz S. (2017). Right Versus Left Colon Cancer Biology: Integrating the Consensus Molecular Subtypes. J. Natl. Compr. Cancer Netw..

[27] Stintzing S., Tejpar S., Gibbs P., Thiebach L., Lenz H.J. (2017). Understanding the role of primary tumour localisation in colorectal cancer treatment and outcomes. Eur. J. Cancer.

[28] Meng C., Bai C., Brown T.D., Hood L.E., Tian Q. (2018). Human Gut Microbiota and Gastrointestinal Cancer. Genom. Proteom. Bioinform..

[29] Lee J.M., Han Y.D., Cho M.S., Hur H., Min B.S., Lee K.Y., Kim N.K. (2019). Impact of tumor sidedness on survival and recurrence patterns in colon cancer patients. Ann. Surg. Treat. Res..

[30] Yang C.Y., Yen M.H., Kiu K.T., Chen Y.T., Chang T.C. (2022). Outcomes of right-sided and left-sided colon cancer after curative resection. Sci. Rep..

[31] Li J., Ma X., Chakravarti D., Shalapour S., DePinho R.A. (2021). Genetic and biological hallmarks of colorectal cancer. Genes Dev..

[32] Arnold M., Sierra M.S., Laversanne M., Soerjomataram I., Jemal A., Bray F. (2017). Global patterns and trends in colorectal cancer incidence and mortality. Gut.

[33] van Dijk S.J., Feskens E.J., Bos M.B., Hoelen D.W., Heijligenberg R., Bromhaar M.G., de Groot L.C., de Vries J.H., Muller M., Afman L.A. (2009). A saturated fatty acid-rich diet induces an obesity-linked proinflammatory gene expression profile in adipose tissue of subjects at risk of metabolic syndrome. Am. J. Clin. Nutr..

[34] Phillips C.M., Kesse-Guyot E., McManus R., Hercberg S., Lairon D., Planells R., Roche H.M. (2012). High dietary saturated fat intake accentuates obesity risk associated with the fat mass and obesity-associated gene in adults. J. Nutr..

[35] Notarnicola M., Miccolis A., Tutino V., Lorusso D., Caruso M.G. (2012). Low levels of lipogenic enzymes in peritumoral adipose tissue of colorectal cancer patients. Lipids.

[36] Kneis B., Wirtz S., Weber K., Denz A., Gittler M., Geppert C., Brunner M., Krautz C., Siebenhuner A.R., Schierwagen R. (2023). Colon Cancer Microbiome Landscaping: Differences in Right- and Left-Sided Colon Cancer and a Tumor Microbiome-Ileal Microbiome Association. Int. J. Mol. Sci..

[37] Xie M.Z., Li J.L., Cai Z.M., Li K.Z., Hu B.L. (2019). Impact of primary colorectal Cancer location on the KRAS status and its prognostic value. BMC Gastroenterol..

[38] Pos O., Styk J., Buglyo G., Zeman M., Lukyova L., Bernatova K., Hrckova Turnova E., Rendek T., Csok A., Repiska V. (2023). Cross-Kingdom Interaction of miRNAs and Gut Microbiota with Non-Invasive Diagnostic and Therapeutic Implications in Colorectal Cancer. Int. J. Mol. Sci..

[39] Ali A., Ara A., Kashyap M.K. (2022). Gut microbiota: Role and Association with Tumorigenesis in Different Malignancies. Mol. Biol. Rep..

[40] Romano G., Veneziano D., Acunzo M., Croce C.M. (2017). Small non-coding RNA and cancer. Carcinogenesis.

[41] Schetter A.J., Okayama H., Harris C.C. (2012). The role of microRNAs in colorectal cancer. Cancer J..

[42] Eneh S., Heikkinen S., Hartikainen J.M., Kuopio T., Mecklin J.P., Kosma V.M., Mannermaa A. (2020). MicroRNAs Associated with Biological Pathways of Left- and Right-sided Colorectal Cancer. Anticancer Res..

[43] Mjelle R., Sjursen W., Thommesen L., Saetrom P., Hofsli E. (2019). Small RNA expression from viruses, bacteria and human miRNAs in colon cancer tissue and its association with microsatellite instability and tumor location. BMC Cancer.

[44] de Miranda N.F., van Dinther M., van den Akker B.E., van Wezel T., ten Dijke P., Morreau H. (2015). Transforming Growth Factor beta Signaling in Colorectal Cancer Cells With Microsatellite Instability Despite Biallelic Mutations in TGFBR2. Gastroenterology.

[45] Johnson S.M., Gulhati P., Rampy B.A., Han Y., Rychahou P.G., Doan H.Q., Weiss H.L., Evers B.M. (2010). Novel expression patterns of PI3K/Akt/mTOR signaling pathway components in colorectal cancer. J. Am. Coll. Surg..

[46] Wautier J.L., Wautier M.P. (2022). Old and New Blood Markers in Human Colorectal Cancer. Int. J. Mol. Sci..

[47] Tutino V., De Nunzio V., Milella R.A., Gasparro M., Cisternino A.M., Gigante I., Lanzilotta E., Iacovazzi P.A., Lippolis A., Lippolis T. (2021). Impact of Fresh Table Grape Intake on Circulating microRNAs Levels in Healthy Subjects: A Significant Modulation of Gastrointestinal Cancer-Related Pathways. Mol. Nutr. Food Res..

[48] Kehl T., Kern F., Backes C., Fehlmann T., Stockel D., Meese E., Lenhof H.P., Keller A. (2020). miRPathDB 2.0: A novel release of the miRNA Pathway Dictionary Database. Nucleic Acids Res..

[49] Brenner H., Kloor M., Pox C.P. (2014). Colorectal cancer. Lancet.

[50] Nagai Y., Kiyomatsu T., Gohda Y., Otani K., Deguchi K., Yamada K. (2021). The primary tumor location in colorectal cancer: A focused review on its impact on surgical management. Glob. Health Med..

[51] Kerr D.J., Domingo E., Kerr R. (2016). Is sidedness prognostically important across all stages of colorectal cancer?. Lancet Oncol..

[52] Ullman T.A., Itzkowitz S.H. (2011). Intestinal inflammation and cancer. Gastroenterology.

[53] Duffy M.J., van Dalen A., Haglund C., Hansson L., Klapdor R., Lamerz R., Nilsson O., Sturgeon C., Topolcan O. (2003). Clinical utility of biochemical markers in colorectal cancer: European Group on Tumour Markers (EGTM) guidelines. Eur. J. Cancer.

[54] Jakus V., Sapak M., Kostolanska J. (2012). Circulating TGF-beta1, glycation, and oxidation in children with diabetes mellitus type 1. Exp. Diabetes Res..

[55] Sheng Y., Li F., Qin Z. (2018). TNF Receptor 2 Makes Tumor Necrosis Factor a Friend of Tumors. Front. Immunol..

[56] Zhang W., Wu N., Li Z., Wang L., Jin J., Zha X.L. (2006). PPARgamma activator rosiglitazone inhibits cell migration via upregulation of PTEN in human hepatocarcinoma cell line BEL-7404. Cancer Biol. Ther..

[57] Moritani K., Hasegawa H., Okabayashi K., Ishii Y., Endo T., Kitagawa Y. (2014). Difference in the recurrence rate between right- and left-sided colon cancer: A 17-year experience at a single institution. Surg. Today.

[58] Bustamante-Lopez L.A., Nahas S.C., Nahas C.S.R., Pinto R.A., Marques C.F.S., Cecconello I. (2019). Is There a Difference between Right- Versus Left-Sided Colon Cancers? Does Side Make Any Difference in Long-Term Follow-Up?. Arq. Bras. Cir. Dig..

[59] Desert R., Giannone F., Schuster C., Baumert T.F. (2023). Tumor microenvironment-derived serum markers as a new frontier of diagnostic and prognostic assessment in biliary tract cancers. Int. J. Cancer.

[60] Hong T.H., Park I.Y. (2014). MicroRNA expression profiling of diagnostic needle aspirates from surgical pancreatic cancer specimens. Ann. Surg. Treat. Res..

